# Suppression of Motor Cortical Excitability in Anesthetized Rats by Low Frequency Repetitive Transcranial Magnetic Stimulation

**DOI:** 10.1371/journal.pone.0091065

**Published:** 2014-03-19

**Authors:** Paul A. Muller, Sameer C. Dhamne, Andrew M. Vahabzadeh-Hagh, Alvaro Pascual-Leone, Frances E. Jensen, Alexander Rotenberg

**Affiliations:** 1 Department of Neurology, Boston Children’s Hospital, Harvard Medical School, Boston, Massachusetts, United States of America; 2 Berenson-Allen Center for Noninvasive Brain Stimulation, Beth Israel Deaconess Medical Center, Harvard Medical School, Boston, Massachusetts, United States of America; 3 Institut Universitari de Neurorehabilitació Guttmann, Universidad Autónoma de Barcelona, Badalona, Spain; 4 Department of Neurology, Perelman School of Medicine, University of Pennsylvania Health System, Philadelphia, Pennsylvania, United States of America; Hospital Nacional de Parapléjicos, Spain

## Abstract

Repetitive transcranial magnetic stimulation (rTMS) is a widely-used method for modulating cortical excitability in humans, by mechanisms thought to involve use-dependent synaptic plasticity. For example, when low frequency rTMS (LF rTMS) is applied over the motor cortex, in humans, it predictably leads to a suppression of the motor evoked potential (MEP), presumably reflecting long-term depression (LTD) – like mechanisms. Yet how closely such rTMS effects actually match LTD is unknown. We therefore sought to (1) reproduce cortico-spinal depression by LF rTMS in rats, (2) establish a reliable animal model for rTMS effects that may enable mechanistic studies, and (3) test whether LTD-like properties are evident in the rat LF rTMS setup. Lateralized MEPs were obtained from anesthetized Long-Evans rats. To test frequency-dependence of LF rTMS, rats underwent rTMS at one of three frequencies, 0.25, 0.5, or 1 Hz. We next tested the dependence of rTMS effects on N-methyl-D-aspartate glutamate receptor (NMDAR), by application of two NMDAR antagonists. We find that 1 Hz rTMS preferentially depresses unilateral MEP in rats, and that this LTD-like effect is blocked by NMDAR antagonists. These are the first electrophysiological data showing depression of cortical excitability following LF rTMS in rats, and the first to demonstrate dependence of this form of cortical plasticity on the NMDAR. We also note that our report is the first to show that the capacity for LTD-type cortical suppression by rTMS is present under barbiturate anesthesia, suggesting that future neuromodulatory rTMS applications under anesthesia may be considered.

## Introduction

Transcranial magnetic stimulation (TMS) is a well-tolerated method for noninvasive stimulation and modulation of regional cortical excitability in humans. TMS is based on the principles of electromagnetic induction where small intracranial electrical currents are induced by a powerful fluctuating extracranial magnetic field. In common clinical and experimental practice, TMS is applied unilaterally over the motor cortex, and coupled with surface electromyography (EMG) such that reliable unilateral motor evoked potentials (MEP) can be recorded from the subject’s contralateral hand muscles. MEP measures can then be used as markers of cortico-spinal excitability [1], [2].

Repetitive transcranial magnetic stimulation (rTMS) of the human motor cortex induces a durable change in cortico-spinal excitability as reflected by a lasting change in the MEP size and appears mediated, at least in part, by intracortical mechanisms [3], [4]. Such capacity to modulate cortical excitability is thought to critically contribute to the therapeutic effects of rTMS in several neuropsychiatric diseases, including major depression, chronic pain and epilepsy [5]–[10].

The mechanisms by which rTMS alters cortico-motor excitability are not sufficiently understood. Human data and experimental work in animals suggest that the lasting effects of high (≥10 Hz) or low (≤1 Hz) rTMS on cortico-spinal excitability rely on synaptic plasticity mechanisms similar to those of long-term-potentiation (LTP) and long-term depression (LTD) [11]–[18]. Specifically, rTMS resembles classical LTD and LTP plasticity in that rTMS effects are frequency dependent, cause an immediate change in excitability, outlast stimulation, and appear to be dependent on activation of the N-methyl-D-aspartate glutamate receptor (NMDAR) [3].

To approximate human protocols in translational (rat) TMS research, our group has developed methods for lateralized single pulse TMS (spTMS) and paired-pulse TMS (ppTMS) to enable focal cortical stimulation and provide a measure of regional cortical excitability [19]–[22]. Here, we establish an rTMS model that will enable mechanistic studies of LF rTMS protocols that are currently used in the clinical arena [23], [24] and anticipate this as a step toward valuable insights at the cellular and molecular level that can be obtained from animal models to improve therapeutic clinical LF rTMS protocols. Specifically, we demonstrate that a lasting reduction in motor excitability can be induced by LF rTMS in anesthetized rats, and examine whether and to what extent the rTMS-induced change in excitability depend on stimulation frequency and the NMDAR.

## Methods

### Ethics Statement

All animal procedures were in accordance with the guidelines of the National Institutes of Health’s *Guide for the Care and Use of Laboratory Animals*. The protocol was approved by the Animal Care and Use Committee at Boston Children’s Hospital (protocol number: 10-03-1633R).

### Animals

48 adult male Long-Evans rats (231 g ±20.7 g) were used. All efforts were made to minimize the number of rats used in the present experiments.

### Anesthesia

Animals were anesthetized with intraperitoneal (i.p.) sodium pentobarbital at 65 mg/kg (50 mg/ml solution). Ten minutes later a second dose, equal to approximately 20% of the primary injection, was given i.p. This pentobarbital dosing protocol was adapted from previously described methods and enabled sufficient anesthesia for the 70-minute experiment [19], [20]. Once anesthetized, rats were placed into a stereotaxic frame (Stoelting Wood Dale, Illinois). The frame was electrically grounded. The stereotaxic frame’s points of contact with the animal (ear bars and nose clamp) were covered with several layers of paraffin film to avoid potential conductance of induced electrical current between the rat and the metal frame, and the rat torso was isolated from the frame base by plastic padding [19].

### Pharmacology

Two separate groups, both stimulated with 1 Hz rTMS, (n = 6 rats/group) received MK801 3 mg/kg (2 mg/ml) or AP5 5 mg/kg (5 mg/ml) dissolved in 0.9% saline by i.p. injection. These doses were based on published methods for systemic administration of the NMDAR antagonists AP5 [25]–[29] and MK801 [30]–[33]. NMDAR antagonists were administered 20 minutes before sodium pentobarbital. Note: due to the sedating effects of MK801, the second dose of pentobarbital was not administered in this group.

### Electromyography

**Figure 1:**
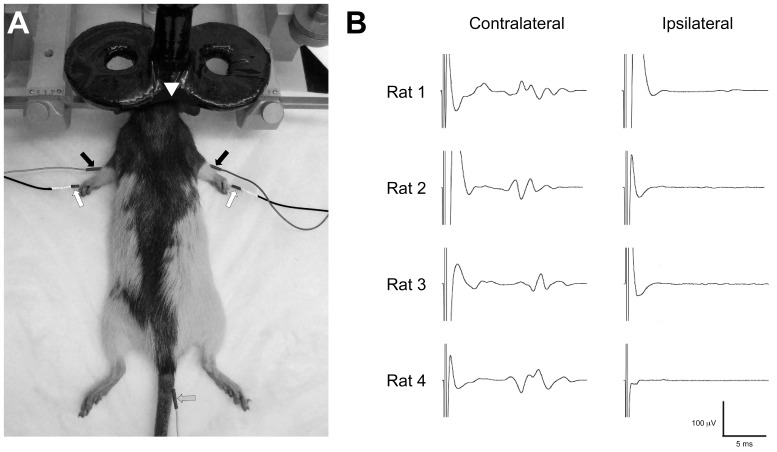
**(A) EMG-rTMS rat setup.** Photograph shows rat in stereotaxic frame. Monopolar stainless steel needle electrodes are placed into the brachioradialis of each forelimb and between the third and fourth digit in the footpad (arrows). A ground electrode is inserted in the tail. A 40 mm figure-of-eight coil is fixed to a micromanipulator arm and positioned over the left or right hemisphere. **(B) Demonstration of contralateral activation by TMS in rat.** Representative brachioradialis MEPs (average of ten consecutive sweeps) are shown. Note, at motor threshold, lateralized TMS elicits isolated MEP in the contralateral forelimb.

MEPs were recorded with monopolar uninsulated 28 G stainless steel needle electrodes (Chalgren Enterprises Inc., Gilroy, CA) inserted into each brachioradialis muscle (Fig. 1a) [19], [20]. Brachioradialis location was determined by palpation of the extended forelimb. A reference electrode was positioned distally in the paw between the 3rd and 4th digit. Each animal was electrically grounded by one needle electrode placed in the tail (Fig. 1a). EMG signal was band pass filtered 100–1000 Hz, amplified X1000 (AM Systems Model 1700; Sequim, WA), digitized with 40 kHz sampling, and stored for post hoc analysis (AD Instruments Colorado Springs, CO).

### Repetitive Transcranial Magnetic Stimulation

A MagStim Rapid stimulator and a customized 70 mm figure-of-eight coil (40 mm external diameter of each lobe; Magstim Company, Wales, U.K.) were used to apply rTMS over the motor cortex (coil center 9.0±0.9 mm lateral and 3.3±1.0 mm anterior to bregma). The coil position was adjusted to produce unilateral MEPs in the brachioradialis muscle contralateral to the site of stimulation and no MEPs in the ipsilateral brachioradialis at motor threshold (MT) (Fig. 1b). By visual inspection, such lateralized TMS produced a twitch only in the contralateral forepaw and shoulder. An exclusive unilateral MEP response was the inclusion criterion for the study.

TMS intensity was recorded as percent machine output (% MO), with 100% corresponding to the maximal electrical current in the coil with corresponding maximal magnetic field. Bregma was located by measurement of the interaural line [34]. MT was defined as the minimum stimulus intensity to obtain MEPs of ≥20 μV peak-to-peak amplitude in at least 5 of 10 trials [20].

To stay within a relatively narrow steady-state anesthesia time window, a pre-rTMS recruitment curve, similar to our previous publication, was rapidly generated by adjusting the stimulator intensity in steps of 5% MO from 55% to 95% MO with a 7 sec inter-stimulus interval for a total of 630 seconds [19]. 10 stimuli were repeated at each step in the recruitment curve. Following baseline and MT acquisition, the stimulator was set to either 100% MT for active stimulation or 5% MO for sham stimulation (n = 6 rats per treatment condition). Stimulation was performed at one of three frequencies; 1 Hz, 0.5 Hz, or 0.25 Hz such that total number of pulses delivered was 300, resulting in 5, 10, and 20 minutes of stimulation for 1, 0.5, and 0.25 Hz, respectively.

**Figure 2:**
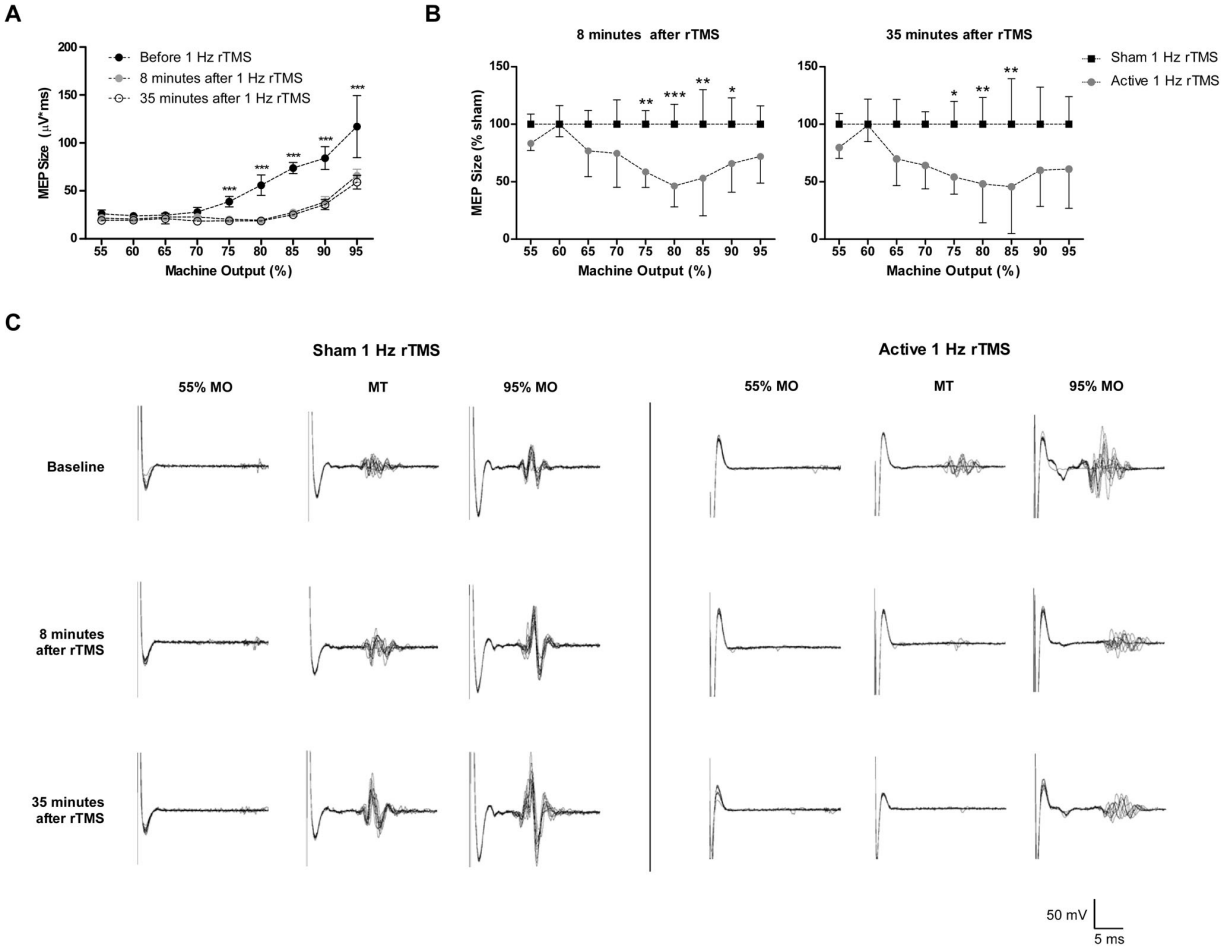
**(A) Representative recruitment curves.** Averaged integrated voltage (uV*s) for the baseline recruitment acquisition from all animals is compared to post-stimulation values at both the 8 and 35-minutes timepoints after rTMS. By 2-way ANOVA, there is a significant reduction in the integrated MEP size following 1 Hz stimulation at both 8-minutes [stimulation effect: F(1,162) = 294.3, p<0.0001; MO effect: F(8,162) = 127.5, p<0.0001; interaction effect: F(8,162) = 25.86, p<0.0001] and the 35-minutes [stimulation effect: F(1,162) = 355.6, p<0.0001; MO effect: F(8,162) = 118.2, p<0.0001; interaction effect: F(8,162) = 27.23, p<0.0001] timepoints. Significance is confirmed by post-hoc t-test from 75–95% MO. Error bars indicate SD. ***p<0.001. **(B) MEP changes following 1 Hz rTMS.** All values are normalized to sham rTMS control. The difference between active and sham rTMS recruitment curves is significant by 2-way ANOVA at the 8-minute [stimulation effect: F(1,90) = 28.35, p<0.0001] and 35-minutes [stimulation effect: F(1,90) = 30.29, p<0.0001] timepoints. At individual stimulator intensities, using post-hoc t-test, there is a significant suppression at 75, 80, 85 and 90% MO (p<0.01, p<0.001, p<0.01, p<0.05, respectively) at the 8-minutes timepoint, and significant suppression at 75, 80 and 85% MO (p<0.05, p<0.01, p<0.01) at the 35-minutes timepoint. Error bars indicate SD. *p<0.05; **p<0.01; ***p<0.001. **(C) Representative MEPs following 1 Hz active or sham rTMS.** Ten consecutive MEP sweeps at 55% MO, 100% MT, and 95% MO from one animal per condition, active or sham rTMS, are shown. Baseline traces are followed by MEPs generated at the same MO at the 8-minutes and 35-minutes timepoints.

For the purposes of timing, we designated end of rTMS as minute “0”. Thereafter, two additional recruitment curves were obtained at 8 and 35 minutes following rTMS (Fig. 2a). The time to the first post-rTMS recruitment curve was limited by the stimulator and coil temperature as pilot runs indicated that a 7-minute cooling period was required for the coil to be functional after 300 rTMS pulses. Therefore, the 8-minute time point was selected as the earliest start point for the recruitment curve used to assess immediate rTMS effects. The second post-rTMS recruitment curve was started 15 minutes after the end of the first post-stimulation curve (lasting 12 minutes), making it the 35^th^ minute of the experiment.

### Statistical Analysis

Absolute integrated voltage from ten consecutive sweeps was averaged for comparison of MEP voltages at each TMS intensity [19], [20]. Means of derived MEP amplitudes were analyzed at each timepoint after LF rTMS as a ratio relative to matched MO from the baseline recruitment curve for each animal. All data expressed as ratios were log-transformed prior to analysis. Group and stimulation intensity contributions to differences between recruitment curves were evaluated by two-way ANOVA with corresponding post hoc paired t-test.

For purposes of analysis, comparisons between multiple groups were made using integrated MEP voltage ratios and their log transformation at 80% MO, an intensity that was the lowest MO value that was suprathreshold for MEP activation in all test animals. We note also that we chose the 80% MO intensity, somewhat arbitrarily, given the relatively coarse MT approximation in 5% MO steps, and absent EMG-based definition of MT in rats.

## Results

### MEP Suppression by LF rTMS

Following 1 Hz rTMS (LF rTMS), the integrated MEP voltage is reduced at all suprathreshold TMS intensities for the duration of the experiment, up to 47 minutes post-rTMS (Fig. 2a). Representative traces demonstrate the reduction seen in MEP amplitude for active 1 Hz stimulation as compared to its sham counterpart (Fig. 2c). Two-way ANOVA comparison of the integrated MEP voltages between groups indicates that recruitment curve is significantly depressed at both the 8-minutes [stimulation effect: F(1,90) = 28.35, p<0.0001] and 35-minutes [stimulation effect: F(1,90) = 30.29, p<0.0001] timepoints following the 300-pulse rTMS train (Fig. 2b). Post hoc t-tests show that the difference is largely attributable to significant suppression at the 8-minutes timepoint for 75, 80, 85 and 90% MO (p<0.01, p<0.001, p<0.01, p<0.05, respectively) when comparing baseline and post-stimulation absolute integrated MEP voltages. For the 35-minutes timepoint, we note a significant decrease in values relative to sham at 75, 80 and 85% MO (p<0.05, p<0.01, p<0.01).

### rTMS Effect is Frequency-dependent

**Figure 3:**
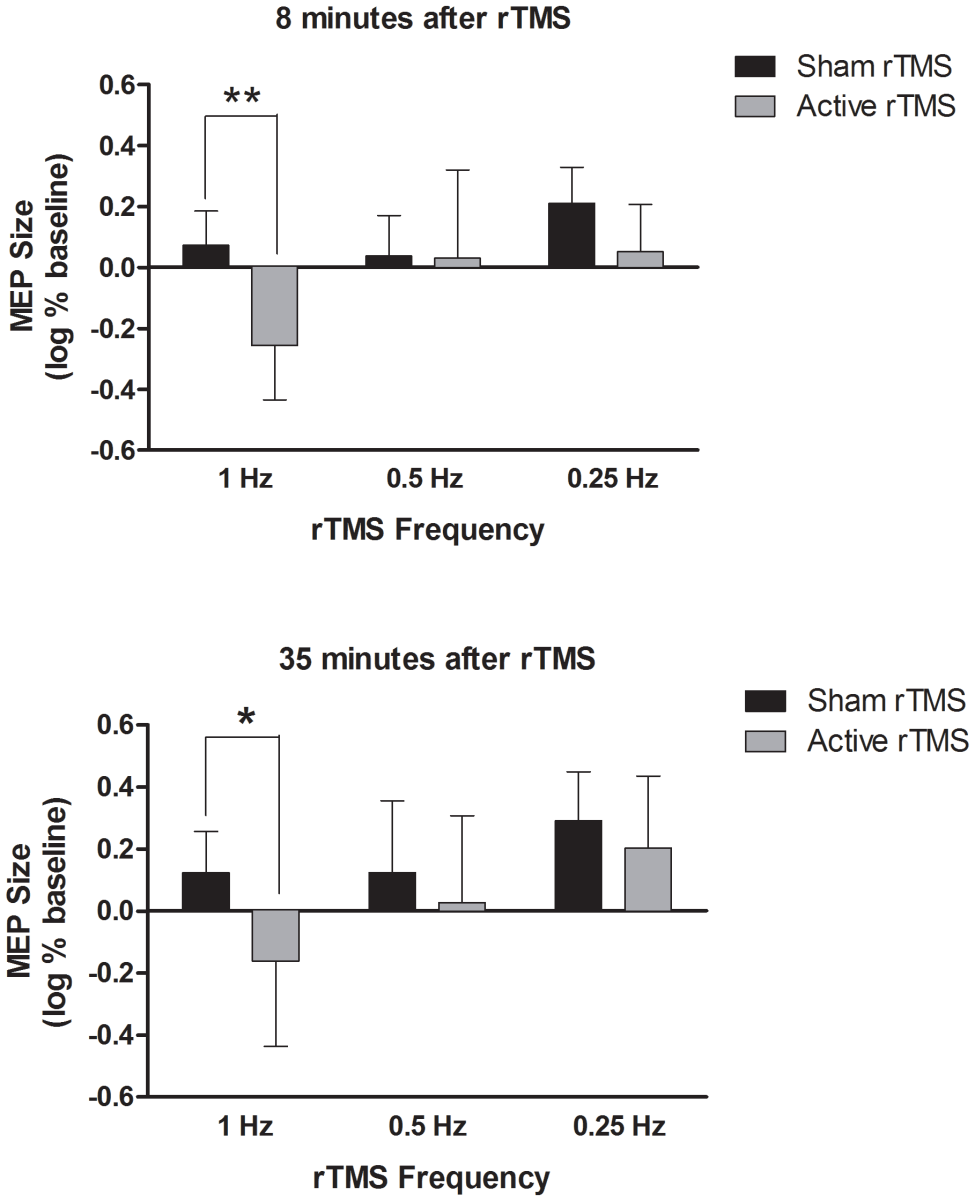
MEP change as a function of rTMS frequency. All conditions are shown at 80% MO. All ratios of average MEP integrated voltage relative to pre-rTMS baseline were log transformed. Compared to its baseline, 1 Hz active rTMS is the only condition where the MEP size was suppressed at both the 8-minues (p<0.01) and 35-minutes (p<0.05) timepoints. Error bars indicate SD. *p<0.05, **p<0.01.

We also find that the suppressive LF rTMS effect is strongly dependent on the rTMS frequency. Specifically, there is no significant difference between sham and active stimulation for either 0.25 Hz or 0.5 Hz at either timepoint after the LF rTMS train. This is illustrated in figure 3 which shows a comparison of the effects of rTMS trains over a short range of frequencies at 80% MO. In contrast, after 1 Hz active rTMS the MEPs are significantly suppressed to 55% (±18%; p<0.004) and 69% (±27%; p<0.04) of the baseline integrated voltage, at the 8-minutes and 35-minutes timepoints, respectively. The time effect not significant for either group, but the frequency effect of frequency was significant for both sham [F(2,30) = 4.43, p<0.021] and active [F(2,30) = 6.20, p<0.0056] by two-way ANOVA. Furthermore, two-way ANOVA analysis of frequency versus stimulation condition at both 8 minute and 35 minutes identified significant contributions of both time points respectively [frequency effect: F(2,30) = 4.88, p<0.015; sham/active effect: F(1,30) = 7.95, p<0.0084] [frequency effect: F(2,30) = 4.31, p<0.023; sham/active effect: F(1,30) = 4.33, p<0.046].

### 1 Hz rTMS Effect is Dependent on NMDAR Activation

**Figure 4:**
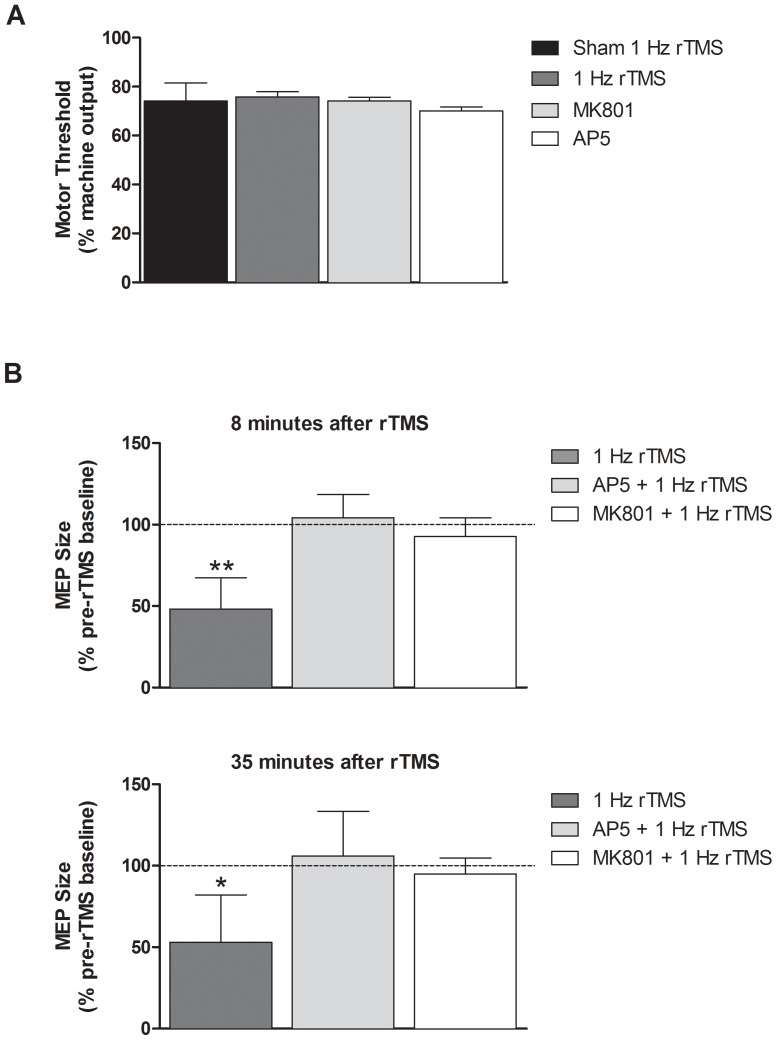
**(A) Average motor threshold per treatment group.** Percent machine output needed to achieve motor thresholds across sham 1(74.2%MO ±7.3%MO, 75.8%MO ±4.9%MO, 74.2%MO ±3.8%MO, 70%MO ±4.5%MO respectively). No significant difference between groups was detected. Error bars indicate SD. **(B) Effects of NMDAR antagonists on MEP suppression by 1 Hz rTMS.** Integrated MEP voltage (uV*s) is measured at 80% MO. Statistics per treatment pair were obtained by comparison with the corresponding sham condition. For the 1 Hz rTMS group, there is a 48.2% (p<0.01) and 53% (p<0.05) reduction at the 8-minutes and 35-minutes timepoints after rTMS, respectively. There is no significant change from pre-rTMS baseline after pretreatment with MK801 or AP5 conditions. Error bars indicate SD.*p<0.05, **p<0.01.

To test whether MEP modulation by LF rTMS is dependent on NMDAR activation, we treated separate groups of rats (n = 6 rats per group) with either the non-competitive NMDA antagonist MK801 or the competitive antagonist AP5. Motor threshold (MT) was unchanged following either MK801 or AP5 administration. MT comparison among treatment conditions showed no significant difference among groups when evaluated by one-way ANOVA (Fig. 4a). Average MT across all groups was 73.5±2.5% MO. Following active rTMS, at 80% MO, we find 48.2% (p<0.01) and 53% (p<0.05) reduction of the MEP integrated voltage at 8 and 35-minutes after the rTMS train, respectively, when compared to their individual pre-rTMS baseline. However, there is no significant difference between sham, MK801, and AP5 conditions (Fig. 4b).

## Discussion

### LTD-like Forelimb MEP Suppression in Anesthetized Rats

As a step toward establishing a mechanistic rTMS model and enabling further trasnslational rTMS research, we show for the first time a long-term depression of the lateralized forelimb MEPs in rats by low frequency rTMS. This lasting modulation of cortico-spinal excitability after rTMS has several essential features of LTD-type plasticity: (1) Frequency dependence with preferential response to 1 Hz stimulation, (2) immediate (within 8 minutes) and durable (up to 35 minutes) modulation of post-stimulation excitability, and (3) NMDAR dependence. Notably, the magnitude and duration of MEP suppression by LF rTMS in our study approximates the 20–50% MEP reduction reported in humans 15–30 minutes after 1 Hz rTMS [13], [35], [36]. We also demonstrate that other aspects of forelimb MEP modulation by LF rTMS are in line with human studies, including frequency dependence and NMDAR involvement. We appreciate that other research groups have also produced analogous results in different experimental rTMS settings [37]–[39], and underscore that the present report adds to the literature results specific to plasticity of a lateralized motor evoked response, akin to that which is seen in humans [19].

### Frequency Dependent Effect of LF rTMS

1 Hz rTMS elicits a greater reduction in motor excitability than either 0.5 Hz or 0.25 Hz stimulation. This is consistent with published LTD data suggesting a preferential response to 1.0 Hz stimulation when compared to other low frequencies [40]. These results also approximate frequency-dependent effects seen in low-frequency rTMS in humans, whereby 1.0 Hz stimulation has the most prominent effect on MEP size [35], [41]. While sub-1.0 Hz stimulation appears to have little to no effect on the MEP in our setup, a depression of the MEP by lower frequencies has been shown in humans [42] and LF rTMS below 1.0 Hz has therapeutic promise in epilepsy (0.3 Hz [43], 0.5 Hz [24]) and tinnitus (0.5 Hz [44]), among other disease states [45], [46]. Plausibly, this discrepancy is due to the physiologic differences between our rat setup and human rTMS, or to the the physiologic effects of anesthesia, or to the relatively short train duration (300 pulses) which is 3–6 times shorter than many human rTMS protocols (reviewed in [42]). Yet an additional and intriguing explanation is perhaps a fundamental difference in physiologic sequelae of 1 Hz rTMS as compared to lower frequencies as suggested in limited human work [41]. Further examination of rodent LF rTMS, particularly if coupled with molecular neuroscience techniques that are available only in preclincal studies, could provide valuable insight into the mechanism of action these sub-1.0 Hz frequencies in neurological disorders. Although detailed study of a large range of stimulation protocols as has been done in humans (reviewed in [42]) is beyond the scope of this experiment, our anticipation is that the present report will facilitate such mechanistic studies with methods for lateralized rTMS in anesthetized rats [19].

### NMDA Dependent Mechanism of Neuronal Modulation by rTMS

Our data provide further insight into LF rTMS mechanism by testing the hypothesis that LF rTMS mediated motor suppression is dependent on NMDAR activation. In human studies, the role of NMDARs in high frequency (potentiating) rTMS has been studied extensively [47], [48]. However, human studies of the NMDAR contribution to motor cortex plasticity with LF rTMS are limited in number, and also limited to only one noncompetitive NMDA antagonist, dextromethorphan, which likely acts on the NMDAR indirectly via active metabolites and may have off-target effects [48], [49]. Similar future human rTMS work with healthy volunteers will also, for ethical reasons, be very likely limited to sub-anesthetic doses of NMDAR antgonists such as ketamine or synthetic opioids. Thus the rat model here provides an explicit validation for the LF rTMS mechanism, as we demonstrate a similar NMDAR dependence in rats as is suggested in human studies, but with more selective NMDAR competitive and noncompetitive NMDAR antagonism.

We chose two common NMDAR antagonists, MK801 and AP5, and found that application of either antagonist prevents MEP depression by 1 Hz rTMS. Notably, in classic in vitro LTD experimental setups, in isolated hippocampal slices, AP5 has been shown to inhibit LTD [50]–[52], but MK801 has not [53], [54]. Thus we find a small discrepancy between the pharmacology of the 1 Hz focal electrical stimulation *in vitro* and 1 Hz rTMS *in vivo*. We underscore this discrepancy as such distinctions in the biochemistry of rTMS effects and those of classic LTD data will need to be reconciled if we are to apply information from the vast LTD literature toward experimental design and clinical rTMS protocols. It is ultimately not surprising that rTMS effects, which could follow from simultaneous electromagnetic stimulation of all components of a cortical volume (principal neurons, interneurons, etc.), may not be fully predicted by data from experimental protocols that involve precise electrical stimulation of a single afferent pathway and focal recording from its target; for instance, where the Schaffer collaterals are stimulated to evoke a post-synaptic response in CA1 in classic LTD experimental setups.

### rTMS in an Anesthetized Subject

Our data are the first to show LTD-type MEP suppression by 1 Hz rTMS in rats under pentobarbital anesthesia. For extension of the present experiment toward further studies, the finding of motor pathway plasticity under anesthesia is valuable, as anesthesia will likely be needed for future rodent rTMS experiments and the interaction between anesthesia and rTMS will require further exploration [20], [55].

An interesting translational extension of the data may be toward testing whether rTMS-mediated cortical plasticity is preserved in humans who are under barbiturate sedation or another anesthetic state. Such scenarios may be of clinical relevance for patient populations, such as children or adults with developmental disabilities, who may benefit from rTMS, but who may not tolerate rTMS in the awake state. Our results demonstrating that cortical excitability can be modulated by rTMS in the anesthetized state also raise the prospect for rTMS in patients treated with high barbiturate doses for management of an acute medical condition such as status epilepticus, as supported by recent case reports [56], [57].

### Study Limitations

We note limitations to the present study that warrant further investigation. Among these, is the confound of anesthesia. As most data in humans are obtained in the waking state, the value of the current findings as a model for human LF rTMS is compromised. The anesthetized state and specific choice of anesthetic likely affect mechanisms of synaptic plasticity as suggested by rodent rTMS results published by Gersner et al. [55]and discussed by Benali et al. [37], and limit extension of the present findings to human rTMS. Along these lines, we cautiously anticipate that the establishment of LTD-like motor depression in rats will enable our lab and others to test the effects of specific anesthetics on rTMS plasticity mechanisms in rodents, in vivo. While the magnitude direction and duration of MEP change after LF-rTMS in rats, as well as its frequency and NMDAR dependence, suggest some mechanistic overlap with human LF rTMS physiology, further experiments to validate these early results, perhaps by ex vivo tissue analysis that follows stimulation of unanesthetized rodents will be needed [38], [58].

Another pharmacologic confound of our experiment, also common to human rTMS studies, is the systemic administration of NMDAR antagonists. While we cautiously infer that the NMDAR dependence of the LTD-like motor phenomenon is cortical, we recognize that systemic drug treatment may affect the corticomotor response at numerous sites between the cortex the neuromuscular junction, as well as at cortical and subcortical motor regulatory systems. Although beyond the scope of this project, further delineation of the contribution of region-specific neurotransmitter receptor populations to rTMS-related cerebral plasticity, as can be accomplished by transgenic or optogenetic methods, or by site-specific microinjections, will be helpful in advancing the value of translational rTMS research [59]–[61].

Last, a necessary limitation of rodent rTMS research is the reduction in TMS spatial resolution. The reduced focality of the electromagnetic stimulus adds to the complexity of the interpretation of TMS effects on distinct neuronal populations that has been discussed in prior translational rTMS studies [37]. Here, as in our prior work, we addressed the problem by eccentric positioning of the coil center relative to the rat’s scalp midline, such that the major coil conductive components are non-tangential to the scalp and thus produce poor electromagnetic coupling between the rat brain and the coil [19], [20], [62], [63]. With this setup, we note that the brachioradialis response was strongly lateralized. However, we recognize that physiologic markers of cortical activation outside the motor areas are absent in our results, and thus interpretation of MEP modulation as resultant from selective motor cortex stimulation should be with caution.

### Conclusion

We provide the first in-vivo electrophysiologic demonstration of LTD-like modulation of motor cortex excitability by low frequency rTMS in anesthetized rats. Consistent with human data, we show effects that outlast stimulation, frequency-dependent cortico-spinal plasticity, as well as a further demonstration of LF rTMS NMDAR-dependence. While limitations of rodent rTMS methods will be essential to address, we are hopeful that our results will facilitate future mechanistic studies, at the cellular and molecular level. Such work is already in progress and that can be best completed in translational rTMS models [37], [55]. We suggest a concerted effort to standardize rTMS studies in animals, with emphasis on specialized coil development. We believe the present study most closely approximates human rTMS findings to this point and expansion of this model will be valuable to uncovering basic rTMS mechanisms as well as to the development of therapeutic rTMS protocols.
